# Surgical Outcomes After Neoadjuvant Chemoimmunotherapy for Resectable Non-Small Cell Lung Cancer

**DOI:** 10.3389/fonc.2021.684070

**Published:** 2021-10-06

**Authors:** Yan Hu, Si-Ying Ren, Ruo-Yao Wang, Chao Zeng, Ji-Na Li, Peng Xiao, Fang Wu, Feng-Lei Yu, Wen-Liang Liu

**Affiliations:** ^1^ Department of Thoracic Surgery, the Second Xiangya Hospital of Central South University, Changsha, China; ^2^ Department of Respiratory and Critical Care Medicine, the Second Xiangya Hospital of Central South University, Changsha, China; ^3^ Department of Cardiothoracic Surgery, the Third Xiangya Hospital of Central South University, Changsha, China; ^4^ Department of Oncology, the Second Xiangya Hospital of Central South University, Changsha, China

**Keywords:** neoadjuvant therapy, immunotherapy, immune checkpoint inhibitors, chemoimmunotherapy, surgery, non-small cell lung cancer

## Abstract

**Background:**

Neoadjuvant chemoimmunotherapy for resectable non-small cell lung cancer (NSCLC) represents an important research topic. Despite the potential benefits of this approach, the inflammatory responses and adverse events associated with neoadjuvant chemoimmunotherapy can present technical challenges and compromise a planned resection. This study assessed the safety and feasibility of neoadjuvant chemoimmunotherapy followed by surgery for resectable NSCLC.

**Methods:**

The study was conducted from May 2019 to March 2021. Patients who were age 18 years or older, were diagnosed with stage Ib–IIIb NSCLC, and received neoadjuvant chemoimmunotherapy followed by surgery were included. Demographic information, clinical and pathologic characteristics, data about neoadjuvant therapy, and surgical details were collected by retrospective chart review. Toxicity profiles were collected retrospectively or by telephone follow-up.

**Results:**

Twenty patients were included in this study. The median age was 56 years (range, 48–72 years), and 18 patients (90%) were men. Squamous carcinoma (14/20, 70%) was the most common cancer type, followed by adenocarcinoma (4/20, 20%), adenosquamous carcinoma (1/20, 5%), and large cell neuroendocrine carcinoma (1/20, 5%). All patients received two to four cycles of neoadjuvant therapy, and the median interval between final therapy and surgery was 49 days (range, 23–133 days). Computed tomography evaluation after neoadjuvant therapy showed partial response in 15 patients (75%) and stable disease in 5 (25%). Final pathologic examinations showed major pathologic response in eight patients, including pathologic complete response in five (25%). Most patients (18/20, 90%) had reduced pathologic staging. Twelve patients (60%) underwent open thoracotomy; the other eight patients underwent minimally invasive surgery, which was uneventful and without intraoperative conversion to open thoracotomy. No perioperative deaths occurred, and only seven patients (35%) developed postoperative complications. Most patients experienced only grade 1–2 adverse effects and laboratory abnormalities during neoadjuvant therapy, and no grade 3 or worse adverse effects or laboratory abnormalities occurred. No patients experienced surgical delays as a result of immune-related adverse events.

**Conclusions:**

Preoperative administration of chemoimmunotherapy for patients with resectable NSCLC was safe and feasible.

## Introduction

Lung cancer is a predominant cause of cancer-related deaths in China and worldwide (1). China reported 733,000 new cases of non-small cell lung cancer (NSCLC) and approximately 610,000 deaths from NSCLC in 2015 (2). NSCLC accounts for more than 85% of all lung cancer cases, of which 30%–40% are resectable cancers, including most stage I–IIIa and a small proportion of stage IIIb lung cancers (3). For early-stage resectable lung cancer, radical surgical resection is the most important therapeutic modality. However, lung cancer is considered a systemic disease; even in early stages, clinically insidious distant organ metastases may already exist. Several studies have reported that approximately 25%–70% of resectable lung cancers will eventually recur, even after complete resection (4). In one study, the 5-year survival rate improved by 5% with platinum-based adjuvant chemotherapy in patients with completely resectable NSCLC (5); however, approximately 20%–30% of patients with stage I, 50% with stage II, and 60% with stage IIIa lung cancers still die within 5 years (3).

In recent years, neoadjuvant therapy has increasingly become a valuable but controversial treatment modality (6). The approach theoretically is effective in reducing tumor size and improving surgical resection rates (7). However, in one study, only a 5% improvement in 5-year survival was obtained with neoadjuvant chemotherapy combined with surgery versus surgery alone for stage Ib–III NSCLC (8). The rate of pathologic complete response (pCR) obtained with neoadjuvant chemotherapy has been only 2%–15% (9, 10). Because targeted therapy and immunotherapy have shown excellent therapeutic promise in metastatic lung cancer, several studies have used them as neoadjuvant therapy for earlier-stage lung cancer (11–13). Unfortunately, phase 3 clinical trials have shown that neoadjuvant targeted therapy did not prolong overall survival: 9.7% of patients achieved major pathologic response (MPR), and no patients achieved pCR, although the progression-free survival benefit of neoadjuvant erlotinib was approximately 10 months longer than survival with neoadjuvant chemotherapy (14). The CheckMate 159 study reported that two doses of neoadjuvant nivolumab was associated with MPR in 9 (45%) of 20 patients with resectable stage I–IIIa NSCLC, and 2 (10%) of the 20 patients achieved pCR (15). A trial combining neoadjuvant atezolizumab with chemotherapy showed that 17 (57%) of 30 patients achieved MPR, and 10 (33%) of the 30 patients achieved pCR (16). Furthermore, for patients with resectable stage IIIa NSCLC enrolled in the NADIM trial, neoadjuvant chemoimmunotherapy was well tolerated and provided a 77% progression-free survival rate and a 90% overall survival rate at 24 months (9).

Although neoadjuvant immunotherapy may have benefits, its mechanism of action raises safety concerns when planning surgical resection (15). It remains unclear whether the inflammatory responses and immune-related adverse events that occur with neoadjuvant immunotherapy can present technical challenges and potentially compromise a planned resection (17). To address this question, this study investigated the safety and feasibility of neoadjuvant chemoimmunotherapy followed by surgery in resectable NSCLC.

## Patients and Methods

This study was conducted at the Second Xiangya Hospital of Central South University and was approved by the Clinical Research Ethics Committee. We retrospectively collected data from May 2019 to March 2021 at our center. Included patients were age 18 years or older, were diagnosed with biopsy-proven clinical stage Ib–IIIb NSCLC, and underwent neoadjuvant chemoimmunotherapy followed by surgery. Patients with unavailable clinicopathological data were excluded. The decision for patients to receive neoadjuvant chemoimmunotherapy was determined jointly by our institution’s surgeons and oncologists in a multidisciplinary discussion mode. Patients underwent laboratory blood tests before each 21-day therapy cycle to monitor complete blood cell counts and biochemical parameters. After a patient received two cycles of neoadjuvant therapy, the treating surgeon evaluated whether the patient was appropriate for surgical intervention or should continue neoadjuvant therapy. All patients underwent standard preoperative staging workup that included pretreatment tumor biopsy, contrast-enhanced computed tomography (CT) scan of the chest, positron emission tomography/CT scan, brain magnetic resonance imaging, and invasive mediastinal nodal staging with endobronchial ultrasound, as indicated. Surgical information—including surgical approach, extent of resection, operative time, estimated blood loss, postoperative hospital length of stay, postoperative complications, reasons for conversion to thoracotomy if necessary, and other information related to the surgical experience, as well as details about the neoadjuvant treatment, including agents, course of treatment, and the interval between the last neoadjuvant treatment and surgery—were collected through the database searches. Toxicity profiles, including adverse events and abnormal laboratory findings, were collected from the database and obtained through additional telephone follow-up if needed.

Tumors were staged according to the 8th edition of the American Joint Committee on Cancer criteria. The presence of comorbidities was assessed using a combination of clinical evaluation and the Charlson comorbidity index (CCI) (18). Adverse events and abnormal laboratory findings were graded according to the National Cancer Institute Common Terminology Criteria for Adverse Events, version 5.0. Surgical complications were assessed according to the Society of Thoracic Surgeons database criteria. MPR and pCR were defined as ≤10% and 0% of viable tumor cells remaining in residual tumor, respectively. Radiologic response assessment was determined according to Response Evaluation Criteria in Solid Tumors.

Continuous variables were expressed as mean ± standard deviation or median and range, as appropriate. Categorical variables were expressed as numbers and percentages.

## Results

### Patient and Cancer Characteristics

From May 2019 to March 2021, 20 patients received neoadjuvant chemotherapy combined with immunotherapy. The median age at diagnosis was 56 years (range, 48–72 years), and 18 patients (90%) were men (**Table 1**). Seventeen patients were current or former smokers. According to the CCI assessment of the presence of comorbidities, 13 patients (65%) had none of the component descriptors (CCI = 0), and the other CCI scores were less common: 6 patients (30%) had a CCI of 1, and 1 patient (5%) had a CCI of 2. One patient underwent gastrectomy followed by adjuvant therapy for gastric adenocarcinoma before the diagnosis of lung cancer. The preoperative clinical staging was as follows: 9 (45%) of 20 had stage IIIa disease; 5 (25%), stage IIIb; 2 (10%), stage IIb; and 1 (5%), stage IIa. Of the histological subtypes, squamous carcinoma (14/20, 70%) was the most common, followed by adenocarcinoma (4/20, 20%), adenosquamous carcinoma (1/20, 5%), and large cell neuroendocrine carcinoma (1/20, 5%). Fourteen patients had tumors exhibiting 1%–50% programmed death ligand 1 (PD-L1) expression, and Ki-67 expression was 30%–70% in most patients (17/20, 85%).

**Table 1:** Patient and cancer characteristics.

Characteristics	
Age, years	
Median (range)	56 (48-72)
Sex	
Female	2 (10)
Male	18 (90)
BMI at presentation, kg/m^2^	
Median (range)	24.2 (18.1–28.9)
Smoking history	
Current/former	17 (85)
Never	3 (15)
CCI	
0	13 (75)
1	6 (30)
≥2	1 (5)
FEV1, percent predicted	
Mean ± SD (range)	89.2 ± 13.1 (60–112)
Clinical stage at presentation	
IIa	
cT2bN0M0	1 (5)
IIb	
cT1cN1M0	2 (10)
IIIa	
cT1aN2M0	1 (5)
cT1bN2M0	2 (10)
cT2aN2M0	1 (5)
cT2bN2M0	2 (10)
cT3N1M0	1 (5)
cT4N0M0	1 (5)
cT4N1M0	1 (5)
IIIb	
cT2bN3M0	1 (5)
cT3N2M0	2 (10)
cT4N2M0	2 (10)
Histologic subtype	
Adenocarcinoma	4 (20)
Squamous cell carcinoma	14 (70)
Adenosquamous carcinoma	1 (5)
Large cell neuroendocrine carcinoma	1 (5)
PD-L1 expression	
<1%	3 (15)
1–50%	14 (70)
>50%	3 (15)

Data are given as number (percentage) unless otherwise indicated. BMI, body mass index; CCI, Charlson comorbidity index; FEV1, forced expiratory volume in 1 s.

### Neoadjuvant Therapy Characteristics

Chemotherapy regimens included 2 patients receiving carboplatin with pemetrexed, 10 receiving carboplatin with paclitaxel, 3 receiving cisplatin with gemcitabine, 2 receiving cisplatin with pemetrexed, and 3 receiving cisplatin with paclitaxel. Sintilimab (7/20, 35%) was the most commonly used immune checkpoint inhibitor, followed by pembrolizumab (6/20, 30%), tislelizumab (5/20, 25%), and toripalimab (2/20, 10%) (**Table 2**). Seven patients (35%) received two courses of preoperative immunotherapy, and seven patients (35%) and six patients (30%) received three and four courses of preoperative immunotherapy, respectively. The median interval of time between the final therapy and surgery was 49 days (range, 23–133 days). CT evaluation after neoadjuvant therapy showed a partial response (PR) in 15 patients (75%) and stable disease (SD) in 5. Final pathologic examinations showed MPR in 8 patients, including pCR in 5 (25%). The majority of patients (18/20, 90%) had reduced pathologic staging.

**Table 2:** Neoadjuvant therapy characteristics.

Characteristics	
Immune checkpoint inhibitor	
Pembrolizumab	6 (30)
Tislelizumab	5 (25)
Sintilimab	7 (35)
Toripalimab	2 (10)
Doses of immunotherapy	
2 doses	7 (35)
3 doses	7 (35)
4 doses	6 (30)
Duration from final therapy to surgery, days	
Median (range)	49 (23-133)
Radiologic response assessment	
PR	15 (75)
SD	5 (25)
Pathologic response assessment	
pCR	5 (25)
MPR	8 (40)
non-MPR	12 (60)
Pathologic downstaging	
Yes	18 (90)
No	2 (10)
Surgical margin	
R0	20 (100)

Data are given as number (percentage) unless otherwise indicated. PR, partial response; SD, stable disease; pCR, pathologic complete response; MPR, major pathologic response.

### Surgical Information of the Included Patients

Twelve patients (60%) underwent open thoracotomy; eight (40%) patients underwent minimally invasive surgery, of whom four underwent robot-assisted thoracoscopic surgery (RATS) and the other four underwent video-assisted thoracoscopic surgery (VATS) (**Table 3**). The patients who underwent minimally invasive surgery all experienced uneventful operations without intraoperative conversion to open thoracotomy. The most common procedure was lobectomy (13/20, 65%), followed by sleeve resection (6/20, 30%) and pneumonectomy (1/20, 5%). One patient underwent bilateral lymph node dissection. The median operative time was 150 min (range, 90–220 min). The median estimated blood loss was 95 ml (range, 60–300 ml). The median postoperative hospital length of stay was 4.9 days (range, 2.5–9.2 days). No patient experienced perioperative death. Seven patients (35%) developed postoperative complications. Pneumonia and urinary retention were the most common complications, both occurring in two patients (10%). One patient underwent reoperation for chylothorax. Atrial fibrillation and hydropneumothorax each occurred in a single patient. One patient who underwent pneumonectomy developed respiratory failure and deep venous thrombosis postoperatively in addition to experiencing urinary retention. One patient who underwent robotic-assisted thoracoscopic lobectomy experienced prolonged air leak (>5 days) in addition to experiencing pneumonia.

**Table 3:** Surgical information of the included patients.

Characteristics	
Surgical approach	
Open	12 (60)
RATS	4 (20)
VATS	4 (20)
Extent of resection	
Lobectomy	13 (65)
Pneumonectomy	1 (5)
Sleeve lobectomy	6 (30)
Unilateral lymphadenectomy	19 (95)
Bilateral lymphadenectomy	1 (5)
Length of postoperative hospitalization, days	
Median (range)	4.9 (2.5–9.2)
Operative time, min	
Median (range)	150 (90–220)
Estimated blood loss, ml	
Median (range)	95 (60–300)
Thirty-day mortality	0
Ninety-day mortality	0
Postoperative complications	7 (35)
Chylothorax	1 (5)
Hydropneumothorax	1 (5)
Respiratory failure	1 (5)
Atrial fibrillation	1 (5)
Thromboembolic event	1 (5)
Pneumonia	2 (10)
Urinary retention	2 (10)
Prolonged air leaks	1 (5)

Data are given as number (percentage) unless otherwise indicated.

### Toxicity Profile of the Included Patients

Thirteen (65%) of 20 patients experienced grade 1 treatment-related adverse effects during neoadjuvant therapy, and 1 (10%) of 20 patients experienced grade 2 adverse effects (**Table 4**). No grade 3 or worse adverse effects occurred. The most common grade 1 adverse effects were alopecia, vomiting, hiccups, and fatigue. One patient presented with grade 2 pruritus, which improved after the application of dexamethasone cream. Seventeen patients had grade 1 laboratory abnormalities. The most common abnormal laboratory findings were anemia, hypoalbuminemia, and elevated blood urea nitrogen. One patient developed grade 2 neutropenia during neoadjuvant therapy, which improved after treatment with recombinant human granulocyte colony-stimulating factor. No patients had grade 3 or worse laboratory abnormalities. No patients experienced surgical delay because of immune-related adverse events.

**Table 4:** Toxicity profile of the included patients.

	Grade 1	Grade 2	Grade 3	Grade 4
Any treatment-related side effects	13 (65%)	1 (5%)	0	0
Hiccup	3 (15%)	0	0	0
Vomiting	4 (20%)	0	0	0
Fatigue	3 (15%)	0	0	0
Nausea	2 (10%)	0	0	0
Pruritus	1 (5%)	1 (5%)	0	0
Rash	1 (5%)	0	0	0
Epistaxis	1 (5%)	0	0	0
Alopecia	7 (35%)	0	0	0
Numbness	2 (10%)	0	0	0
Anorexia	1 (5)	0	0	0
				
Any treatment-related abnormal laboratory findings	17 (85%)	1 (5%)	0	0
Anemia	13 (65%)	0	0	0
Eosinophilia	3 (15%)	0	0	0
Neutropenia	1 (5%)	1 (5%)	0	0
Hypothrombocytopemia	3 (15%)	0	0	0
Hypoalbuminemia	7 (35%)	0	0	0
Increased aminotransferases	4 (20%)	0	0	0
Hyperbilirubinemia	1 (5%)	0	0	0
Increased blood urea nitrogen	5 (25%)	0	0	0
Increased uric acid	4 (20%)	0	0	0
Increased proBNP	0	0	0	0
Hypocalcemia	2 (10%)	0	0	0

Data are given as number (percentage) unless otherwise indicated.

## Discussion

In this small study, the use of neoadjuvant chemotherapy with immunotherapy before surgical resection for patients with resectable NSCLC was safe and feasible.

As the efficacy of immunotherapy in advanced or metastatic NSCLC has been demonstrated (19, 20), attention has naturally turned toward the use of programmed cell death receptor-1 (PD-1)/PD-L1 inhibitors in early-stage NSCLC (21). Immune checkpoint inhibitors act by blocking immunosuppressive PD-1/PD-L1 interactions to activate T cells and kill tumor cells (22). The application of immunotherapy in the neoadjuvant setting has several theoretical advantages. First, the increased number of activated tumor-specific CD8+ T cells resulting from the presence of the primary tumor releases more tumor neoantigens when killing tumor cells, and these antigens are presented to intratumor-specific effector T cells at different sites—including the primary site, metastases, and the circulatory system—to activate a broad and powerful specific antitumor immune response and immune memory. Second, the relatively intact structure of the lymphatic system provides great opportunities for tumor cell–immune cell interactions. Third, the efficacy of neoadjuvant therapy can be evaluated directly from the resected specimen (21). Thus, one may hypothesize that preoperative use of immunotherapy is superior to postoperative use in improving long-term prognosis and eradicating distant metastases. Furthermore, a preclinical study has confirmed that mice inoculated with metastatic breast cancer cells and treated with neoadjuvant anti-PD-1 have increased numbers of tumor-specific CD8+ T cells in the peripheral blood and organs, and they experience a longer survival period than their counterparts treated with adjuvant anti–PD-1 (23).

MPR and pCR are excellent surrogates for overall survival in chemotherapy-related clinical trials and are now the most commonly used indicators to assess pathologic response to neoadjuvant immunotherapy. Junker et al. (24) developed and refined the criteria for grading of tumor regression after use of chemoradiation in the neoadjuvant setting and found that therapy-induced tumor regression of less than 10% viable tumor tissue is associated with improved long-term prognosis in NSCLC. However, the antitumor tissue response evoked by immunotherapy should differ from the response to neoadjuvant chemoradiation (25). Therefore, pathologic response criteria designed for chemotherapy and radiotherapy may not be suitable for use with neoadjuvant immunotherapy (25). It remains unclear whether MPR and pCR can be used as valid surrogate markers for neoadjuvant immunotherapy. Optimal thresholds of pathologic response suggestive of a good prognosis also must be explored in future studies.

A series of clinical trials used neoadjuvant immunotherapy in resectable NSCLC (17). Forde et al. (15) were the first to prospectively investigate the safety and feasibility of neoadjuvant immunotherapy in resectable NSCLC. They reported that preoperative administration of two courses of nivolumab was associated with few side effects, did not cause a delay in surgery, and resulted in MPR in 9 (45%) of 20 patients with resectable lung cancer (15). The phase 2 LCMC3 study also showed that preoperative administration of two cycles of atezolizumab induced SD in 89% of patients, PR in 7%, MPR in 18%, and pCR in 5%. In a phase 1b trial in patients with stage Ia–IIIb disease receiving neoadjuvant sintilimab, Gao et al. showed that 40.5% of patients achieved MPR and 16.2% achieved pCR (26). Several trials of neoadjuvant chemoimmunotherapy have been designed to increase response rates and improve prognoses (9, 16). In a phase 2 trial, Provencio et al. (9) reported that 83% of 41 patients with stage IIIa NSCLC achieved MPR, 71% achieved pCR, and no patients had delayed surgery because of neoadjuvant therapy. In another phase 2 trial, Shu et al. (16) demonstrated that neoadjuvant atezolizumab and chemotherapy induced MPR in 57% of patients with stage Ib–IIIa NSCLC. Recently, Jiang et al. (26) retrospectively analyzed the surgical perspective with neoadjuvant immunotherapy in resectable NSCLC and found that 12 (38.7%) of 31 patients had MPR and 3 (9.6%) had pCR. This study included 20 patients with stage IIa–IIIb NSCLC who received neoadjuvant chemoimmunotherapy. Our initial results showed that 40% achieved MPR and 25% achieved a pCR, and both results were comparable to those in previous studies.

Although neoadjuvant immunotherapy has attracted great interest in the treatment of early-stage resectable NSCLC, it is paramount to consider intraoperative technical difficulties and the potential for adverse drug events during or after treatment (21). In a phase 1 trial of neoadjuvant nivolumab for resectable NSCLC, Bott et al. (13) reported no intraoperative deaths but found that 10 (50%) of 20 patients developed perioperative complications, including atrial fibrillation (6/20, 30%) as the most common complication. Moreover, 7 (54%) of 13 patients who underwent minimally invasive surgery experienced conversion to open thoracotomy (13). In the NADIM trial, Provencio et al. (9) reported that 14 (30%) of 46 patients experienced grade 3 or worse treatment-related adverse reactions and that the most common reactions were increased lipases (3/46, 7%) and febrile neutropenia (3/46, 7%). However, none of these treatment-related adverse effects resulted in delayed surgery or death (9). In a prospective study, Yang et al. (27) found no apparent difference in perioperative morbidity and mortality rates between patients receiving neoadjuvant chemotherapy plus ipilimumab and patients receiving neoadjuvant chemotherapy alone before surgery for stage II–IIIa NSCLC. Our initial results demonstrated that the majority of patients experienced only grade 1–2 adverse effects and laboratory abnormalities during neoadjuvant therapy and that no grade 3 or worse adverse effects or laboratory abnormalities occurred in the study population. In our study, no patient experienced a delay in surgery or perioperative death. Only seven patients (35%) developed postoperative complications. These complication rates were considered acceptable; they were comparable with the results of previous studies using a neoadjuvant cohort (13, 27, 28) and with the results of studies without a neoadjuvant cohort as 42.5% (29). It seems that patients can safely undergo operations even after neoadjuvant chemoimmunotherapy.

Operative time, blood loss, and conversion to open thoracotomy are other indicators of the difficulty of surgery. Our study reported operative times comparable to published results (28) and low intraoperative bleeding rates. Although most patients underwent open surgery, no conversion to open thoracotomy was needed for those who initially received minimally invasive surgery. This finding suggests that neoadjuvant chemoimmunotherapy did not increase the surgical complexity. Because the number of patients in our study was limited, a larger sample size study is needed to validate the results.

The optimal course of neoadjuvant immunotherapy and the optimal interval between the last therapy and surgery are poorly defined. Using results from available phase 1/2 clinical trials, expert consensus recommends that two to four doses of preoperative neoadjuvant immunotherapy is appropriate and that surgery can be performed 4–6 weeks after the last therapy (30). In our study, all 20 patients received two to four cycles of neoadjuvant therapy and had a median interval between therapy and surgery of 49 days. Our findings can provide a reference for clinical practice in the absence of current large-volume trial evidence.

This study had several limitations. By design, the study involved a single center and was retrospective. Selection bias may have existed. In addition, the number of patients included in this study was very small, and we did not collect prognostic information on these patients.

In conclusion, the use of neoadjuvant chemoimmunotherapy before surgical resection for patients with resectable NSCLC was safe and feasible. Future large-scale studies that include long-term follow-up data are required to verify these outcomes.

## Data Availability Statement

The raw data supporting the conclusions of this article will be made available by the authors, without undue reservation.

## Ethics Statement

The studies involving human participants were reviewed and approved by the Ethics Committee of the Second Xiangya Hospital, Central South University, Changsha. The patients/participants provided their written informed consent to participate in this study.

## Author Contributions

YH and W-LL were involved in the design of the study. YH, S-YR, and R-YW were involved in data acquisition. YH, S-YR, R-YW, CZ, J-NL, PX, FW, F-LY, and W-LL were involved in data analysis and interpretation. YH and W-LL were involved in manuscript preparation. All authors contributed to the article and approved the submitted version.

## Funding

This work was supported by the National Natural Science Foundation of China (81972638) and the Natural Science Foundation of Hunan Province, China (2019JJ30038).

## Conflict of Interest

The authors declare that the research was conducted in the absence of any commercial or financial relationships that could be construed as a potential conflict of interest.

## Publisher’s Note

All claims expressed in this article are solely those of the authors and do not necessarily represent those of their affiliated organizations, or those of the publisher, the editors and the reviewers. Any product that may be evaluated in this article, or claim that may be made by its manufacturer, is not guaranteed or endorsed by the publisher.
